# Growth in Children After a Kidney Transplant: A Retrospective, Observational Single-Center Study

**DOI:** 10.7759/cureus.69003

**Published:** 2024-09-09

**Authors:** Mugahid Elamin, Bashair Alabbasi, Majed Aloufi

**Affiliations:** 1 Pediatric Nephrology, Prince Sultan Military Medical City, Riyadh, SAU

**Keywords:** children, eskd, growth, height, kidney transplant, weight

## Abstract

Background: Kidney transplantation (KTX) is the best treatment for children with end-stage kidney disease (ESKD). It greatly improves their quality of life. Children's growth is one of the chronic issues that is known to be compromised during ESKD; therefore, catch-up growth is usually expected to be seen after KTX.

Objectives: We aimed to evaluate children's catchup growth after KTX and assess the impact of children's age at the time of KTX on catchup growth.

Patients and methods: We performed a retrospective analysis of weight and height data for children pre-KTX, at 12 months, and 24 months post KTX. We stratified them into five percentile categories for weight and height and counted the number of KTX patients in each category at the same three time points. We also stratified them into three different age groups: two to five, six to 10, and 11 to 12 years, and estimated the mean and standard deviation of both weight and height of each one.

Results: Between 2009 and 2019, we identified 37 children who underwent KTX. The mean weight pre-KTX was 21 kg. It increased to 28 and 34 kg post KTX at 12 and 24 months, respectively. The mean height pre-KTX was 115 cm. It increased to 126 and 134 cm post KTX at 12 and 24 months, respectively. There was a significant crossing of both weight and height percentiles when we stratified them based on different initial percentiles. There was a significant change in both weight and height when we stratified them into three age groups: two to five, six to 10, and 11 to 14 years.

Conclusion: The growth patterns of children after a KTX can vary among children. However, our retrospective observational study showed positive results, suggesting gradual improvement in weight and height gain post KTX. Factors such as age at the time of KTX, duration of kidney disease, medication regimens, and overall health status can influence a child's growth trajectory. Close monitoring, proper nutrition, and a multidisciplinary approach are essential in supporting a child's growth after a KTX. Our findings are limited by the small sample size and retrospective design, therefore a well-structured prospective study with a large sample size is required.

## Introduction

Growth is significantly compromised in children with chronic kidney disease (CKD) secondary to a variety of factors such as uremia, malnutrition, metabolic derangements, metabolic bone mineral disorders, as well as abnormal growth hormone. Kidney transplantation (KTX) is currently the best treatment for children with end-stage kidney disease (ESKD). Overall health, well-being, and quality of life are among the main goals of KTX in children with CKD. The final height achieved in adulthood has a significant impact on the medical, psychological, and social aspects of these patients. Growth is an important indicator of health in children with CKD. Therefore, it seems intuitive to expect growth improvement after KTX. Unfortunately, optimal growth cannot always be achieved even with a well-functioning allograft. Optimizing growth prior to KTX, early detection of growth retardation, and timely intervention are important factors [1].

Many factors have been found to affect post-KTX growth. Among those, the age of children at the time KTX proved crucial for accelerated post-KTX growth. Many KTX centers found that KTX in the age range between two and five years has a significant effect on catch-up growth [2]. Furthermore, the first five years following KTX, when the kidney allograft functions are greatest, showed remarkable catch-up growth, with the peak growth rates occurring within the initial two years post KTX. Despite that, it has been noticed that a few numbers of school-age children and adolescents fail to experience any catch-up growth, and some of them encounter even a decline in growth instead. Not only that, but the severity of the height deficit pre-KTX is a pivotal determinant of post-KTX catch-up growth, with children exhibiting severe height deficits at the time of KTX having greater potential for catch-up growth. Therefore, optimizing the nutritional status in children with CKD is an essential factor to prioritize during each follow-up clinic visit [1, 3]. Corticosteroids are another important player; their use has a significant impact on the prevalence of stunting growth following KTX, and children who had received immunosuppression regimens to completely withdraw from or avoid corticosteroids showed a remarkable catch-up growth [1,4].

The aim of this study is to assess the growth changes in children after KTX at 12 and 24 months, as well as to address the impact of the age of children at the time of KTX on catch-up growth.

## Materials and methods

Study population

Our study is a single-center retrospective study in children less than 15 years of age who underwent KTX between 2009 and 2019 in the pediatric kidney transplantation unit at Prince Sultan Military Medical City (PSMMC), Riyadh, Saudi Arabia. Data were collected from the patient's medical records. We included children whose weight and height measurements were available at the time of KTX and at 12 and 24 months after KTX. A total of 37 children were enrolled. None of the included children had been diagnosed to have short stature secondary to non-CKD-related conditions, nor have skeletal abnormalities that may compromise expected normal height. As per international standard guidelines, the study received approval by the Institutional Review Board at PSMMC.

Inclusion criteria

Both genders, male and female, from the ages of two to 14 years with kidney failure, were enrolled. We included children whose weight and height measurements were available at the time of KTX and at 12 and 24 months after KTX. Hemodialysis, peritoneal dialysis, and preemptive patients were included. In addition, the enrollment included deceased donors, living-related donors, and non-related donors.

Exclusion criteria

Children with acute kidney injury and other organ failure were excluded. None of the included children have been diagnosed to have short stature secondary to non-CKD-related conditions, neither have skeletal abnormalities that may compromise expected normal height, and none of them have chronic use of immunosuppression medication before the kidney transplant.

Data collection

We collected the baseline characteristics of all children enrolled in the study, including sex, age at KTX, the primary cause of CKD, type of dialysis before KTX, type of KTX, type of induction immunosuppression, type of maintenance immunosuppression, the status of allograft function at 12 and 24 months after KTX, as well as weight and height at three time points, before KTX, 12 and 24 months after KTX. We also collected whether there was growth hormone utilization before KTX or not.

Statistical analysis

Because of the small sample size and the descriptive retrospective design of our study, we chose descriptive statistics including the mean, standard deviation, average, and percentage to describe the baseline demographics and the frequency of occurrence of independent and dependent variables. For better clarification of our dependent variables, we stratified our cohort based on weight and height percentiles into five categories and calculated the number of KTX patients in each category at three time points before KTX, 12 and 24 months after KTX, in order to have a deeper look at the effects of KTX in each category. We also stratified our cohort based on three age categories: two to five years, six to 10 years, and 11 to 14 years, and estimated the mean and standard deviation of both weight and height for each category, in order to assess the differences in catch-up growth between different age groups.

## Results

Children who underwent KTX in Prince Sultan Military Medical City between 2009 and 2019 were included in this study. Twenty-three of them were male (62.2%) and 14 were female (37.8%). The age group of the study participants was between two and 14 years with six patients (16.2%), 14 patients (37.8%), and 17 patients (45.1%), from two to five years, six to 10, and 11 to 14, respectively. Seventeen (45.1%) of them were on hemodialysis (HD), 18 (48.7%) were on peritoneal dialysis (PD), and only two (5.4%) were preemptive KTX. There were 26 (70.3%) deceased donors (DD KTX), while living related donors (LRD KTX) and living unrelated donors (LURD KTX) were six (16.2%) and five (13.5%), respectively. Growth hormone was used by six patients (16.2%) before KTX. Induction using anti-thymocyte globulin (ATG) was used for 29 recipients (78.4%), while basiliximab was used as induction for eight (21.7%). Most of our use was for mycophenolate mofetil, and we only faced very minor problems in terms of abdominal pain and diarrhea in general where the percentage of patients was estimated at 20% only. We used mycophenolate sodium as a replacement and did well with them, so the symptoms varied according to different patients. None of the patients used chronic use of immunosuppression medication before the kidney transplant and were on triple immunosuppression (37, 100%) are shown in Tables 1-2, respectively.

**Table 1:** Baseline patients' characteristics HD: hemodialysis; PD: protein dialysis; PKT: preemptive kidney transplant; DD: deceased donors; LRD: living related donors; LURD: living related donors; ATG: anti-thymocyte globulin

Variables	Description	Frequency (N) Percentage (%)
Gender	Male	23 (62.2%)
	Female	14 (37.8%)
Age groups (years)	2 to 5	6 (16.2%)
	6 to 10	14 (37.8%)
	11 to 14	17 (45.1%)
Type of dialysis	HD	17 (45.1%)
	PD	18 (48.7%)
	PKT	2 (5.4%)
Type of kidney transplant	DD	26 (70.3%)
	LRD	6 (16.2%)
	LURD	5 (13.5%)
Small for gestational age	No	37 (100%)
	Yes	0 (0%)
Growth hormone before kidney transplant	No	31 (83.8%)
	Yes	6 (16.2%)
Induction of Immunosuppression medication	Methylprednisolone/ATG ‎	29 (78.4%)
	Methylprednisolone/Basiliximab ‎	8 (21.6%)
Maintenance Immunosuppression medications	Double Immunosuppression	0 (0%)
	Triple Immunosuppression	37 (100%)

**Table 2:** Immunosuppression drug dosages protocol post kidney transplantation Note: This table represents medications as the protocol for all pediatric kidney transplant recipients [5]. *Start mycophenolate mofetil one day before transplantation at 600 mg/m2/dose twice daily; reduce the dose to 300 mg/m2/day twice daily once a target tacrolimus level is achieved. ** Tacrolimus is to be started once allografts function adequately, serum creatinine is less than 250 umol/l, or drops by more than 50% from the baseline. Adjust dosage based on target level.

Medication	Dose	Frequency
Anti-thymocyte (mg/kg) for deceased kidney transplant, Day 0, 1, 2, and 3	4.5	Accumulative dose
Basiliximab (mg/kg) for living kidney transplant, Day 0 and 4	<35 kg: 10mg, >35 kg: 20 mg	2 doses
Mycophenolate* (mg/m2)	600 then 300	Every 12 hours
Tacrolimus** (mg/kg/day) start from Day 1	0.3	Every 12 hours
Predinsilone protocol		
Time post transplant	(mg/m2)	Frequency
Day 0	200	As induction then after 6 hours
Time post transplant	(mg/kg/day)	Frequency
Day 1	2.00	every 6 hours
Day 2	1.75	every 6 hours
Day 3	1.50	every 6 hours
Day 4-6	1.25	every 12 hours
Day 7-9	1.00	every 12 hours
Day 10-12	0.75	every 12 hours
Day 13-14	0.50	every 12 hours
Week 3	0.45	Once/day
Week 4	0.40	Once/day
Week 5	0.30	Once/day
Week 6-7	0.25	Once/day
Week 8-9	0.20	Once/day
Week 10-12	0.15	Once/day
Week 13-16	0.10	Once/day
Week >16	0.10 mg/kg/dose	Once/day

The mean weight at the time of KTX was 21.3 kgs (standard deviation (SD) 7.4), which increased significantly during the follow-up period after 12 and 24 months to a mean weight of 27.9 kgs (SD 11.2) and 33.8 kgs (SD 14.1), respectively. The mean height at the time of KTX was 115.2 cms (SD 17), which increased significantly during the follow-up period after 12 and 24 months to a mean height of 125.5 cms (SD 15.8) and 134.4 (SD 16.2), respectively (Table 3).

**Table 3:** Mean and SD of weight and height at three time points KTX: kidney transplantation

Time of transplant	Weight (kgs)		Height (cms)	
	Mean	Std. deviation	Mean	Std. deviation
Pre-KTX	21.3	7.4	115.2	17
After 12 months	27.9	11.2	125.5	15.8
After 24 months	33.8	14.9	134.4	16.2

**Figure 1:**
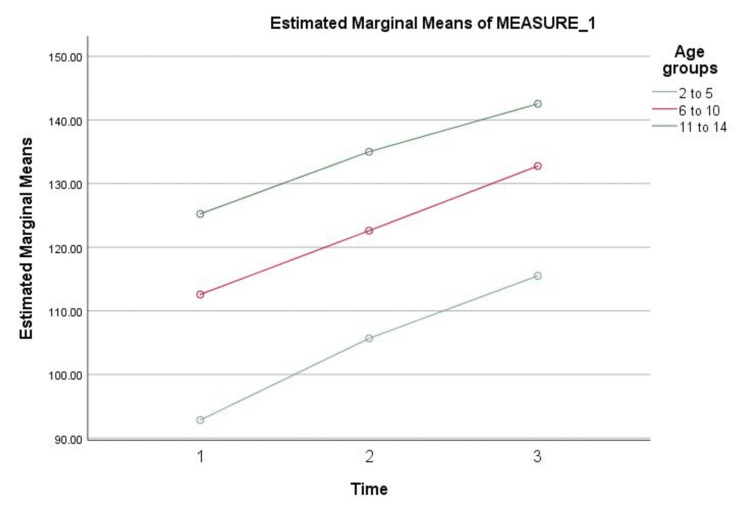
Changes in weight and height of three age categories at three time points in order to assess the differences in catch-up growth between different age groups (in years)

We further stratified our cohort into three age categories as mentioned earlier, and the result of the mean and SD of each category before KTX, at 12 months, and 24 months after KTX is shown in (Table 4) and (Figure 1), which showed significant changes in both weight and height to assess the differences in Catch up growth between different age groups.

**Table 4:** Mean and SD of weight and height stratified into three age categories at three time points KTX: kidney transplantation

Time	Age groups (years)	Weight (kgs)			Height (cms)		
		Mean	Std. deviation	N	Mean	Std. deviation	N
Pre-KTX	2 to 5	12.6	2.6	6	92.8	8.6	6
	6 to 10	20.2	5.3	14	112.6	10.6	14
	11 to 14	25.3	7.2	17	125.2	15.4	17
	Total	21.3	7.4	37	115.2	17	37
After 12 months	2 to 5	15.9	4.1	6	105.7	7.4	6
	6 to 10	27.4	4.8	14	122.6	8.8	14
	11 to 14	32.5	13.5	17	135	15.2	17
	Total	27.9	11.2	37	125.5	15.8	37
After 24 months	2 to 5	18.7	3.4	6	115.5	9.6	6
	6 to 10	33.1	6.6	14	132.8	9.9	14
	11 to 14	39.7	18.4	17	142.5	16.5	17
	Total	33.8	14.9	37	134.4	16.2	37

We stratified our cohort one time more based on their corresponding weight and height percentile into five categories: less than 5^th^ percentile, between 5^th^ and 10^th^ percentile, more than 10^th^ and 25^th^ percentile, more than 25^th^ and 50^th^ percentile, and more than 50^th^ percentile. Again, we used the same three time points. Overall, the results were remarkable for significant increments in both weight and height, as shown in Tables 5-6, respectively. For example, there were 24 patients (64.7%) of our cohort less than the 5^th^ percentile for weight pre-KTX, while only 12 patients (32.4%) and seven patients (18.9%) continued to be less than the 5^th^ percentile at 12 and 24 months post KTX, respectively. There were only three patients (8.1%) more than the 50^th^ percentile pre-KTX; these numbers increased to six patients (16.2%) and nine patients (24.3%) that were more than the 50^th^ percentile at 12 and 24 months post KTX, respectively (Table 4). A similar finding has been observed with height, as there were 27 patients (72.1%) of our cohort less than the 5^th^ percentile for height pre-KTX, while only 17 patients (45.1%) and 11 patients (29.7%) continued to be less than the 5^th^ percentile at 12 and 24 months post KTX, respectively. There was only one patient (2.7%) more than the 50^th^ percentile pre-KTX, while there were one patient (2.7%) and seven patients (18.9%) that were more than the 50^th^ percentile at 12 and 24 months post KTX, respectively (Table 6).

**Table 5:** Number of patients (percentage) for each weight percentile category at three time points KTX: kidney transplantation

Weight percentile	Before KTX	After 12 months	After 24 months
‎<5^th^ percentile‎	24 (64.7%)	12 (32.4%)	7 (18.9%)
5^th^ -10^th^ percentile‎	5 (13.5%)	4 (10.8%)	5 (13.5%)
>10^th^-25^th^ percentile‎	4 (10.8%)	13 (35.1%)	10 (27%)
>25^th^-50^th^ percentile‎	1 (2.7%)	2 (5.4%)	6 (16.2%)
>50^th^ percentile‎	3 (8.1%)	6 (16.2%)	9 (24.3%)

**Table 6:** Number of patients (percentage) for each height percentile category at three time points

Height percentile	Before the transplant	After 12 months	After 24 months
‎<3^rd^ percentile‎	27 (72.1%)	17 (45.1%)	11 (29.7%)
‎3^rd^-10^th^ ‎percentile‎	3 (8.1%)	7 (18.9%)	7 (18.9%)
‎>10^th^-25^th^ percentile‎	4 (10.8%)	7 (18.9%)	6 (16.2%)
‎>25^th^-50^th^ percentile‎‎	2 (5.4%)	5 (13.5%)	6 (16.2%)
‎>50^th^ percentile‎	1 (2.7%)	1 (2.7%)	7 (18.1%)

## Discussion

Children grow primarily during their early years, and their growth potential decelerates shortly after puberty. Nutritional, genetic, and hormonal factors play integral roles in determining a child's growth potential [6]. Chronic kidney disease greatly compromises the normal growth of children. Successful KTX in children is known to improve the growth pattern, especially with well-functioning kidney allografts; although the growth rate varied, the overall trend showed positive results [7]. Various factors can influence a child's growth after a KTX. These factors include the age of the child at the time of KTX, the duration of CKD before KTX, medication regimens, particularly steroid use as immunosuppression drugs, and overall health status post KTX. Therefore, it is essential for healthcare providers to carefully monitor children's growth and adjust medication dosages if necessary [8]. Ensuring optimal growth after KTX involves a multidisciplinary approach. This includes close coordination between the KTX team, nutritionists, and the child's family. Regular monitoring of growth charts and frequent follow-up visits play a crucial role in identifying any growth-related concerns and taking appropriate action. Nutrition also plays a vital role in supporting a child's growth after a KTX; a well-balanced diet rich in essential nutrients is essential. The child's healthcare team can guide on appropriate calorie intake, protein requirements, and vitamin supplementation if needed [9].

Our current retrospective study focused on assessing the growth patterns of children under 15 years of age who underwent KTX. The study aimed to analyze the changes in weight and height measurements of the pediatric recipients before KTX, 12 months post KTX, and 24 months post KTX. The mean weight of the children at the time of KTX showed a significant increase at 12 and 24 months post KTX. Similarly, the mean height also displayed a noticeable growth trend over the same follow-up periods. Stratification of the cohort by age, weight, and height percentiles further highlighted the substantial improvements in both weight and height post KTX. The findings of this study demonstrate the positive effects of KTX on the growth parameters of pediatric recipients. The significant increments in weight and height measurements indicate the successful impact of KTX on the overall growth of children with ESKD. This study sheds light on the promising outcomes of KTX in promoting growth in children with CKD.

Different reports and studies have addressed the growth of children after KTX with variable outcomes. In the annual 2014 North American Pediatric Renal Transplant Cooperative Study (NAPRTCS) report, there were no remarkable height increments post KTX [10]. In the European Renal Association/European Dialysis and Transplantation Association (ESPN/ERA-EDTA) report, normal heights were reached in 55% of children after KTX [11]. A research group from Barcelona has found an improvement in linear growth after a median follow-up of 38 months, with a significant baseline growth impairment and greatest height changes having been detected in children younger than three years in comparison to negligible changes in older children [12]. Another study from Germany found a significant increase in mean height velocity as well as height within a couple of years after KTX in children that received a well-functioning kidney allograft before the beginning of puberty [13]. Another recent study from Turkey focused on the longitudinal growth of prepubertal children in the first two years post KTX. Thirty-four prepubertal children, with the majority being boys, underwent KTX at a mean age of 7.3 years. They conclude that KTX can have a positive impact on linear growth in prepubertal children, which indicates that KTX can lead to improved linear growth. Interestingly, the study also found a negative correlation between the mean age of the children at the time of KTX and their height standard deviation scores, which means that younger children tend to show greater improvements in growth following KTX [14]. Overall, the findings of these studies have more or less comparable finding to the results of our study.

Ultimately, it is clear that KTX not only improves the quality of life but also plays a crucial role in promoting growth and development in pediatric patients. By emphasizing personalized care and monitoring growth parameters post-KTX, healthcare providers can ensure the best possible outcomes for children undergoing KTX.

## Conclusions

The growth patterns of children after a KTX can vary among children. However, our retrospective observational study showed positive results, suggesting gradual improvement in weight and height gain post-KTX. Factors such as age at the time of KTX, duration of kidney disease, medication regimens, and overall health status can influence a child's growth trajectory. Close monitoring, proper nutrition, and a multidisciplinary approach are essential in supporting a child's growth after a KTX. Our findings are limited by the small sample size and retrospective design, therefore a well-structured prospective study with a large sample size is required.
